# Human Papillomavirus Distribution in Women with Abnormal Pap Smear and/or Cervical Intraepithelial Neoplasia in Vaccination Era. A Single-Center Study in the North Italian Population

**DOI:** 10.3390/microorganisms9040729

**Published:** 2021-03-31

**Authors:** Barbara Gardella, Mattia Dominoni, Cecilia Sosso, Anna Arrigo, Andrea Gritti, Stefania Cesari, Giacomo Fiandrino, Arsenio Spinillo

**Affiliations:** 1 Department of Obstetrics and Gynecology, Fondazione IRCCS Policlinico San Matteo, University of Pavia, 27100 Pavia, Italy; cecilia.sosso@gmail.com (C.S.); anna_arrigo@hotmail.com (A.A.); andreagritti1205@gmail.com (A.G.); spinillo@smatteo.pv.it (A.S.); 2 Department of Clinical, Surgical, Diagnostic and Paediatric Sciences, University of Pavia, 27100 Pavia, Italy; matti.domino@gmail.com; 3 Department of Pathology, Fondazione IRCCS Policlinico San Matteo, University of Pavia, 27100 Pavia, Italy; s.cesari@smatteo.pv.it (S.C.); g.fiandrino@smatteo.pv.it (G.F.)

**Keywords:** cervical intraepithelial neoplasia, human papillomavirus, pap smear, vaccination

## Abstract

Time trends prevalence of human papillomavirus (HPV) genotypes including negative and untypable infections were analyzed during a 15-year period (2005–2019) among 5807 subjects with abnormal pap-smears and/or cervical intraepithelial neoplasia (CIN). The rates of HPV16 dropped by 13% every 3 years (Prevalence Ratio, PR = 0.87, 95% CI = 0.82–0.93) in the CIN1 biopsy, while HPV16 status was unchanged over time in the CIN2+ biopsy. In CIN1 lesions, there was a corresponding increase of HR-HPV types unrelated to nonavalent vaccine. The rates of HPV 18, 31, and 52, decreased by 35% (PR = 0.65, 95% CI = 0.54–0.79), 19% (PR = 0.81, 95% CI = 0.73–0.91), and 21% (PR = 0.79, 95% CI = 0.73–0.86) every 3-year interval in CIN2+, respectively. Overall, the prevalence of negative/untypable HPV specimens in the entire database increased from 9.6% (129/1349) in the period 2011–2013 to 17.6% (161/913) and 28.4% (224/790) in the 2014–2016 period and in the 2017–2019 period, respectively (PR = 1.69, 95% CI = 1.52–1.88). HPV 16 prevalence decreased significantly among subjects with low-grade cervical squamous lesions. A significant increase of both HPV types unrelated to nonavalent vaccination and negative/untypable HPV infections was reported. The prevalence of HPV types among subjects with abnormal pap smears in Northern Italy is changing. Many variables including demographic factors and possibly vaccination could be responsible for this modification.

## 1. Introduction

Persistent high-risk human papillomavirus (HPV) infection, mostly caused by HPV 16 and HPV 18, is the leading cause of invasive cervical cancer [1]. The most effective strategy for cervical cancer prevention is vaccination against HR-HPV in young females. According to the literature data, a decrease of HR-HPV genotypes is expected over the next ten years, after the introduction of the vaccination program, but data to evaluate the relationship between HPV vaccination and the subsequent risk of invasive cervical cancer are lacking [2].

Before the introduction of the vaccination program in Italy, the distribution of HPV types in CIN lesions over the period 1985–2007 in Northern Italy changed significantly as a consequence of the introduction of new HPV types from other geographical areas and/or modifications of social and behavioral variables [3]. In particular, HPV types 51, 52, 53, 56, and 58, that were uncommon 20 to 30 years ago are now found in over one third of Cervical Intraepithelial Neoplasia (CIN) [3]. Moreover, HPV 6, 11, 16, and 18, actually targeted by available multivalent vaccines, were two to three times less frequent around 2000s than in the previous two decades [3]. The reduction of HPV 16 and HPV 18 infections especially in younger women in cervical specimens obtained from screening has been noted even in recent years in the U.S. [4] and has been attributed to vaccination and to the presence of emerging HPV types (especially 83 and 61). Based on data of European meta analysis, HPV 16 and/or 18, although with some differences between Northern and Southern countries, are estimated to be responsible for 52–76% of all cases of high grade cervical lesions [5]. Early results on the relationship between vaccination and HPV-related subsequent lesions in the Italian population confirm that HPV-16 related CIN after vaccination is a rare event confirming a probable change, current and future, in the HPV genotype distribution in preinvasive cervical lesions [6].

Although currently available HPV genotyping assays are very accurate in the identification of HPV types with proven and probable carcinogenicity, 1–2% of cervical samples shows a positive signal in HPV lines but fails to show a positive signal in subsequent HPV typing [7,8,9]. These untypable HPV infections, also called HPV X, are mainly caused by HPVs 83, 42, 81, 67, 90, 74, 87 and others, and are associated with an increased risk of CIN [7,8]. While data on the trends over time of HPV 16 and other carcinogenic HPV types in different geographic areas are increasingly studied, little or no data on the trends over time of HPV X in cervical lesions are available. The purpose of this study was to evaluate the trends of HPV genotypes including negative and untypable HPVs over 15 years in cytological cervical specimens of women attending colposcopic procedures due to an abnormal pap smear.

## 2. Materials and Methods

Data for this study were extracted from a database containing prospectively collected clinical, colposcopic, and virological information from subjects aged 21 to 65 years of age attending the Colposcopic Service of our Department due to an abnormal screening pap smear. For the purpose of the study, we evaluated 5 consecutive 3-year periods from 2005 to 2019.

Subjects were referred by the cytological screening service of our department and external institutions and from private practice. Exclusion criteria included age <21 years, ongoing pregnancy, history of positive HPV test or treatment for CIN or total hysterectomy before enrolment, and non-squamous lesions on pap smear or with preinvasive or invasive lesions. Institutional Review Board (IRB) approval by our institution was obtained (RC805036 IRCCS Fondazione Policlinico San Matteo, Pavia, Italy).

The database was composed by a series of anamnestic items compiled after structured interviews at entry and by clinical, colposcopic, and virological items compiled at entry and during the follow-up. All patients were treated according to an established protocol including HPV DNA detection and genotyping and colposcopy with targeted biopsies. Cervical samples for HPV genotyping were obtained immediately before colposcopy. After speculum examination, scrapes were taken with a cervix brush, suspended in ThinPrep-PreservCyt Solution (Cytic Corporation, Marlborough, MA, USA), and stored at 4 °C. DNA extraction was performed by lysis and digestion with proteinase K. HPV sequences from the L1 region were amplified by means of the polymerase chain reaction (PCR) using SPF10 primers in a 50 μL final reaction volume for 40 cycles. Appropriate positive and negative controls were introduced for each set of reactions. Concurrent amplification of beta globin sequences was used as a control for DNA adequacy. HPV type-specific sequences were detected by the line probe assay INNO-LiPA HPV genotyping assay version V2 up to 2009 and version EXTRA subsequently (Fujirebio Europe. Gent, Belgium), according to the manufacturer’s instructions. Hybridization patterns were automatically analyzed by the LiRAS system and checked by two independent readers. The risk of the HPV type associated with the development of cancer is based on the data of the International Agency for Research on Cancer (IARC).

For the purpose of this study, we classified HPV types into four categories: high risk with proven carcinogenicity targeted with novalent HPV vaccines (Group 1: 16, 18, 31, 33, 45, 52, 58), high risk HPVs not targeted with nonavalent vaccines (Group 2: 26, 35, 39, 51, 53, 56, 59, 66, 68, 73, 82), Low risk HPVs (Group 3: 6, 11, 40, 43, 44, 54, 69, 70, 74), and HPV untypable (HPV positive signal for generic probes and negative for genotyping essays) [10].

A standardized colposcopic examination was performed immediately after cervical brushing for HPV genotyping by two different gynecologists (BG, MD) certified by the Italian Society of Colposcopy. Multiple targeted cervical biopsies were obtained in all cases where CIN2+ was suspected on colposcopy and in all cases of high-grade squamous cervical lesions (HSIL) irrespective of colposcopic impression. Endocervical curettage was performed, according to the clinician’s judgment, when the extent of the lesion or the squamocolumnar junction was not entirely visible (NTZ Type 3) or in the case of atypical glandular cells (AGC) on Pap smear. Histological diagnoses were based on consensus decision of two expert gynaecological pathologists (CS, FG). In the analysis of the data, we either used the histological diagnosis of punch biopsy or, in more severe cases, the diagnosis was determined after cone biopsy obtained by the loop electro-excision procedure (LEEP) or by cold knife excision.

Univariate statistical analysis was carried-out with Kruskal–Wallis analysis of variance and chi-square test to compare continuous and categorical variables, respectively. The Bonferroni method for multiple comparisons was used to evaluate partitioned chi-square tests in multiway contingency tables. Chi-square for trend was used to test for univariate linear trend. Adjusted prevalences and prevalence ratios (PR) were obtained by using the Poisson regression model including the different types of HPV infections as outcomes and age upon examination, HIV status (yes, no), nulliparity (yes, no), ancestry (Italian, European, others), and multiple HPV infections (yes, no) as confounding variables. All the analyses were carried out with STATA 13.0 [11].

## 3. Results

Out of 5864 subjects between 21 and 65 years of age attending the colposcopy service of our department in the 2005–2019 period and potentially eligible for this study, we excluded 57 (1%) subjects because colposcopy was impossible and/or unsatisfactory, leaving 5807 women for the final analysis. Unfortunately, full data about HPV vaccination in naïve subjects was available only in a small number of subjects referred to our department, so inferences about the role of vaccination upon HPV type distribution cannot be made. Table 1 reports the main demographic characteristics of the subjects upon enrolment in each three-year period. Tests for trends suggested that the median age at examination and the rates of extra-European ancestry increased, whereas the rates of HIV-positivity, multiparity and multiple HPV infection decreased significantly during the period of the study.

In Table 2, we summarized the colposcopic, virological, cytological, and histological findings in five periods under study. The overall rates of CIN1 and CIN 2+ on the entire database were 27% (1566/5807) and 16.3% (2459/5807). During the period of the study, the rates of larger (>50% of the ectocervix) lesions increased over the time, whereas the prevalence of multiple HPV infections decreased. After adjustment for confounders, the prevalence ratio of multiple infections decreased significantly every three years both in ASCUS/LSIL (PR = 0.78, 95% CI = 0.75–0.81) and HSIL (PR = 0.82, 95% CI = 0.75–0.91) lesions. The overall distribution of cytological and histological diagnoses were heterogeneous across the period of time examined as demonstrated by the overall chi square analysis (chi-square = 112.6, *p* < 0.001 and chi-square = 88.8, *p* < 0.001, respectively). Regarding the types of HPV detected over time, although the frequencies of the different categories were heterogeneous (chi-square = 556, *p* < 0.001), there was a significant reduction in the rates of LR-HPV and an increase in HR-HPV unrelated to nonavalent vaccine genotypes in the last 3–6 years compared to previous periods. Overall, the tests for trends also confirmed an increase in the rates of HR-HPVs unrelated to vaccination, negative and untypable HPVs. After adjustment for potential confounders, the increase of the three-year prevalence ratio of HR-HPV unrelated to nonavalent vaccination was homogeneous across ages at examination strata being 1.21 (95% CI = 1.1–1.33), 1.16 (95% CI = 1.1–1.26), and 1.17 (1.05–1.31) among women of 21–29, 30–45 and 45–65 years, respectively. In the 2011–2019 period, after adjustment for potential confounders the prevalence ratios of negative and untypable HPVs increased by 1.53 (95% CI = 1.36–1.72) and 2 (1.78–2.36) times every three-years, respectively.

To evaluate the potential displacement of HPV types, we studied the distribution over time of the 7 HR-HPVs (16, 18, 31, 33, 45, 52, 58) targeted by nonavalent vaccines both among cytological (Table 3) and histological (Table 4) diagnoses. Overall, after adjustment for confounders, in the entire database there was a reduction of HPV 16 infection by about 6% every three years (PR = 0.94, 95% CI = 0.89–0.98). In adjusted Poisson analysis, the drop was significant among single (PR = 0.87, 95% CI = 0.82–0.91) but not in multiple (PR = 1.1, 95% CI = 0.98–1.23) HPV infections. In addition, when the analysis was stratified for age (21–29, 30–45, 45–65 years) HPV16 infection dropped only among young women (21–29) (PR = 0.84, 95% CI = 0.77–0.92). The adjusted prevalence ratios of HPV 16 (PR = 0.91, 95% CI = 0.87–0.95), 18 (PR = 0.82, 95% CI = 0.73–0.91), 31 (PR = 0.86, 95% CI = 0.79–0.92), 52 (0.91, 95% CI = 0.86–0.95) dropped over time among women with ASCUS/LSIL on pap smear upon entry but not among HSIL subjects (Table 3). Moreover, the age effect was also evident in the subgroup of subjects with ACUS/LSIL; in fact, the adjusted prevalence of HPV16 dropped only among younger women (21–29 years) (PR = 0.83, 95% CI = 0.76–0.92) but not in the age group 30–45 (PR = 0.95, 95% CI = 0.68–1.33) or 46–65 (PR = 0.98, 95% CI = 0.88–1.08). Finally, in the entire database, the reduction of HPV 16 infection was confirmed among young women (21–29) of Italian ancestry (PR = 0.84, 95% CI = 0.76–0.93) but not among foreigners (PR = 0.85, 95% CI = 0.67–1.07).

When the analysis was restricted to the 3475 women who had a positive colposcopically-directed biopsy (Table 4), the rates of HPV 16 and HPV 31 dropped by 13% and 16% every 3 years (PR = 0.87, 95% CI = 0.82–0.93 and PR = 0.84, 95% CI = 0.75–0.94, respectively) among women with CIN1 biopsy results. Even in this case, after adjustment for confounders, the drop in the rate of infection was significant among women of 21–29 years of age (PR = 0.8, 95% CI = 0.69–0.73) but not among subjects aged 30–45 (PR = 0.9, 95% CI = 0.8–1.02) or 46–65 (PR = 0.94, 95% CI = 0.83–1.07). Among subjects with CIN2+ results on biopsy/cone specimens, the overall rates of HPV 16 rates were unchanged over the time of observation. Finally, in CIN2+ subjects the rates of HPV 18, 31, and 52, decreased by 35% (PR = 0.65, 95% CI = 0.55–0.78), 19% (PR = 0.81, 95% CI = 0.73–0.91), and 21% (PR = 0.79, 95% CI = 0.73–0.86) every 3-year interval.

**Figure 1:**
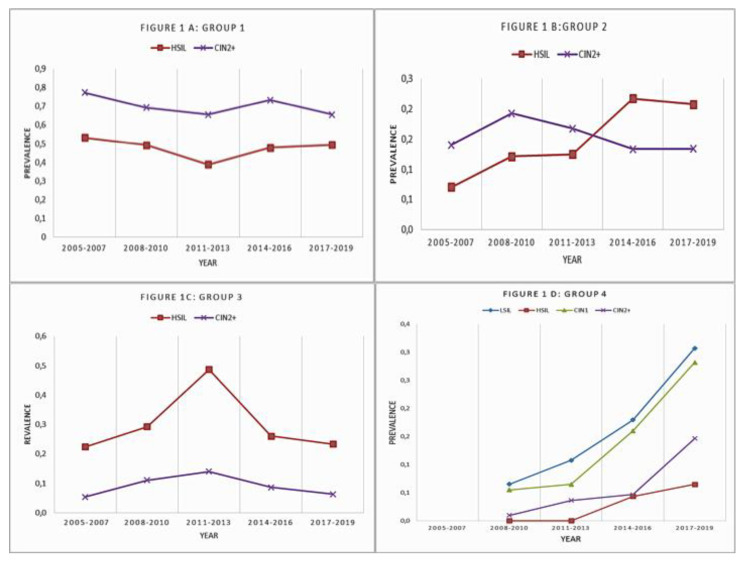
Association between cytological and histological findings and human papillomavirus (HPV) genotypes. (**A**) prevalence of the seven High risk-HPV related to nona-valent vaccine on cytology and histology. (**B**) prevalence of the other HR-HPVs not included in vaccine on cytology and histology. (**C**) prevalence of the low-risk HPV on cytology and histology. (**D**) prevalence of the negative/untypable HPV on cytology and histology.

The overall prevalence of the seven HR-HPVs targeted by nonavalent HPV vaccines were substantially unchanged during the period of the study (PR = 1, 95% CI = 0.94, 1.07) irrespective of the severity of cervical lesions and age category (Figure 1A). 

The rates of HR-HPVs unrelated to nonavalent vaccines increased every three-year period among subjects with ASCUS/LSIL (PR = 1.12, 95% CI = 1.06–1.18), HSIL (PR = 1.28, 95% CI = 1.1–1.52), CIN1 lesions on biopsy (PR = 1.2, 95% CI = 1.12, 1.28) but not among subjects with CIN2+ histological diagnosis (PR = 0.96, 95% CI = 0.86, 1.07) (Figure 1B).

The rates of LR-HPVs (Figure 1C) decreased over time only among subjects with ASCUS-LSIL (PR = 0.91, 95% CI = 0.89–0.95) or CIN1 lesions on biopsy (PR = 0.89, 95% CI = 0.85–0.94). 

Overall, the prevalence of negative/untypable HPV specimens in the entire database increased from 9.6% (129/1349) in the 2011–2013 period to 17.6% (161/913) and 28.4% (224/790) in the 2014–2016 period and 2017–2019, respectively (PR = 1.69, 95% CI = 1.52–1.88) (Figure 1D.) Among subjects with histological CIN 1, the prevalence of negative/untypable HPV was 6.7% (35/525), 15.6% (59/378) and 27.9% (101/362) in the 2011–2013, 2014–2016, and 2017–2019 periods, respectively (PR = 1.9 (95% CI = 1.64–2.17).

The corresponding rates of negative/untypable HPV in the same periods among CIN2+ subjects were 3.7% (8/214), 4.1% (6/141) and 14.6% (22/151) (PR = 2.12, 95% CI = 1.49–3.02).

Finally, in the 2011–2019 period in the overall database, the increase in the prevalence ratio of negative/untypable HPV was homogeneous across age at examination strata being 1.9 (95% CI = 1.6–2.3), 1.76 (95% CI = 1.54–2) and 1.6 (95% CI = 1.36–1.87) at 21–29, 30–45 and 45–65 years, respectively.

## 4. Discussion

The distribution of HPV types involved in cervical infection depends on the characteristics of the population, including geographical area, presence and severity of lesions and age. In the present study, during 15 years, the rates of HPV 16 and other HR-HPVs such as HPV-18, 31, 52, 58 dropped significantly among subjects with low grade lesions. On the other hand, among subjects with CIN2+ on biopsy the prevalence of HPV 16 and other HR-HPV related to nonavalent vaccines remained stable during the period of observation. The behavior of HR-HPV unrelated to nonavalent vaccines increased correspondingly in low risk cervical lesions mirroring the behavior of HPV16 infection suggesting a potential replacement of HPV16 by other HR-HPVs. Finally, the prevalence of the specimens negative for HPV or with untypable HPV infections increased consistently in the 2011–2019 period, irrespective of the severity of cervical lesions and age category.

The variation in the rates of single HPVs associated with cervical lesions during different periods of time could be influenced by several factors including age, a proxy for sexual activity and HPV exposure, parity and race, prevalence of other sexually transmitted diseases and severity of cervical lesions in the population studied [12]. In addition, the interference between the different HPV types in multiple infections and the effect of the introduction of HPV vaccination in the population studied could heavily influence the incidence of single HR-HPV infections [4,13]. 

A significant reduction in the prevalence of HPV-16 related cervical lesions in the last 10 years, especially among low grade lesions and severe lesions among younger women, has been already reported by other authors [4,14]. Similarly, to our analysis, time trends of severe cervical lesions caused by HPV 16 remained stable suggesting that an epidemiological displacement of this type of virus is probably recent and will be evidently severe in the following years. Interestingly, in our study, the drop in the prevalence of HPV-16 related lesions was accompanied also by a reduction of the prevalence of HPV 18 and HPV 31 and by a corresponding increase of HR-HPVs unrelated to nonavalent vaccines in low risk lesions and in high risk lesions of young (21–29 years) women. Since the natural history of CIN lesions is well known, and low-grade squamous lesions usually predate high grade and invasive lesions by many years, it is possible to speculate that the increased rate of HR-HPVs unrelated to vaccine is a relatively recent trend and it is mainly evident in new, incidental low grade squamous lesions and high-grade lesions of young women [15]. 

The biological reasons for the reduction in the rates of HPV 16 infection and the corresponding increase in the rates of HR-HPVs are various. Although in stratified analysis, the modifications in the rates of HR-HPVs were confirmed only in the groups of young women of Italian ancestry, the increase in the rates of foreign women attending our colposcopic service during the 15 years studied suggests that immigration could play a significant role in the relative prevalence of HR-HPVs by introducing new HPV types in the population. Several studies support this view suggesting that ‘new’ HPV types are very common among immigrants in Italy [16].

Multiple HR-HPV infections are rather common, involving from 10–40% of HPV infections [17,18]. Several studies have shown that in multiple infections, HPV 16 could interact with other HR-HPVs influencing their frequency [17,19]. In the present study, the rates of HPV16 cervical infections dropped almost exclusively among subjects with a single HPV 16 infection suggesting that the number of infecting HPVs did not influence the reduction in the rates of HPV 16 infection. Another possible explanation for the modifications of HPvs in cervical lesions over time is the introduction of vaccines in the population. Hariri et al. [20] found that in a population-based sentinel surveillance system in USA the incidence of HPV 16 and 18 high grade cervical lesions dropped significantly after the introduction of HPV vaccination. Similar results have been reported in cervical screening programs in Europe suggesting that the introduction of a quadrivalent vaccine is associated with a reduction of HPV 16, 18, 31, 33, and 45 due to cross-protection [21]. Since in this study we have no complete data on vaccination because vaccinated women will start cervical cancer screening program in 2022, we cannot make inferences about the direct effect of vaccines on HPV prevalence for the following years. Nevertheless, Bogani et al. [6] in a recent multicenter study on Italian women suggest that high grade CIN occurring after HPV vaccination is a rare condition. Other potential biases of the study include the single center nature of the recruitment. This could have introduced some forms of unmeasurable biases. Finally, HPV data were obtained in a selected group of women attending colposcopy because of an abnormal pap smear and the corresponding results cannot be automatically translated in general population. Although factors such as race, country of origin, and other behavioral factors could contribute to the introduction of ‘new’ types of HPVs among people at risk, [5,12,16,21] the prevalence, incidence and oncogenic role of untypable HPVs in general population remain to be elucidated. 

However, most of our data suggest that a reduction of the rates of HPV 16 and other HR-HPVs related to vaccination among women with abnormal pap smears, and conversely the increase in HR-HPVs unrelated to vaccination, was mainly found among young women with low grade lesions. This is in agreement with a population study of HPV infection in the general population carried out in self-collected cervico-vaginal samples [22]; in this study, the rates of HPV related to quadrivalent vaccines dropped significantly only among women up to 24 years of age remaining stable thereafter. In Italy, HPV vaccination was introduced in 2007 and is free for female adolescents; the current vaccination coverage is about 60–70% of the female population [23]. Based on the coverage data, it is possible that displacement of HPV 16 and other HR-HPVs related to vaccines in this study could be the result of vaccine-related immunity.

The increase in the rates of HPV negative and HPV untypable specimens during the period of 2010–2019 in our study is puzzling. As reported by several studies, the prevalence of HPV negative specimens among women with abnormal pap smears, CIN or invasive cervical cancer is about 12–14% [24,25], a rate that is comparable to our data. Interestingly Petry et al. reported that HPV-negative status in cervical cancer could be a false diagnosis or false-negative HR-HPV results, caused by use of HC2 method [26].

From a methodological viewpoint, the INNO-Lipa extra system is considered a reliable method for genotyping HPVs when compared to hybrid capture methods [27], although the analytical sensitivity is slightly lower than that of the original SPF10-LIPA25 probe [28]. On the other hand, guidelines for HPV DNA testing requirements in cervical cancer screening cautioned against the use of tests with excessive sensitivity because a small increase in sensitivity could result in a significant increase of false positives [29]. On the other hand, an in-depth analysis of HPV-negative specimens with additional alternative procedures such as procedures targeting E6/E7 showed that almost half of negative HPV specimens are in fact positive for HR-PVs [26,30]. HPV negative results have also been attributed to the presence of small lesions shedding few abnormal cells, or also too early or too late, healing infections shedding few virus copies [24].

Untypable HPV infection is defined as having positive PCR results using consensus primers (SPF10) but a failed positive signal on genotyping assays [7,8]. This type of infection is associated with an intermediate risk of CIN [7,8]. Molet et al. have studied with a high-throughput sequencing of HPV variables showing that more than 40% of HPV-X samples are in fact infected by uncommon HPVs and the remaining untypable HPVs could be the results of HPV quasispecies infections (so-called variants) whose oncological potential is uncertain [8]. The prevalence of untypable HPV infection is about 3–4% in invasive cervical cancer but is much higher in ASCUS/LSIL cytological samples [31]. In the literature, there are no data on the time trends of HPV negative or HPV untypable infections on cervical samples. 

In our study, the increased prevalence of HPV negative or HPV untypable specimens was confirmed in CIN 1 and CIN2+ histological lesions and was independent of age suggesting that the relative modifications of the frequencies of HPV types in cervical samples recorded in recent years involve not only HPV unrelated to vaccines but also to uncommon HPV types, or, possibly, to HPV quasispecies [8].

A definite approach including virological, cytological and histological homogeneous data during a 15-year period is the main strength of the study. On the other hand, data were limited only to abnormal pap smears and were collected in a single center; for these reasons, our results cannot be applied to general population. 

## 5. Conclusions

In conclusion, during a 15-year period there were significant modifications in the prevalence of different types of HPVs among women with abnormal pap smears. The reduction in the rates of HPV 16, 18 and other HR-HPVs was mirrored by an increase in the rates of HPVs unrelated to vaccines and by HPV negative/untypable specimens.

## Figures and Tables

**Table 1:** Demographical and clinical characteristics of women enrolled in the five periods under study.

	2005–2007 N = 1163 (%)	2008–2010 N = 1592 (%)	2011–2013 N = 1349 (%)	2014–2016 N = 913 (%)	2017–2019 N = 790 (%)
Age (years)					
21–29	333 (28.6)	374 (23.5)	341 (25.3)	256 (28)	206 (26.1)
30–45	590 (50.7)	848 (53.3)	680 (50.4)	402 (44)	341 (43.2)
>45	240 (20.6)	370 (23.2)	328 (24.3)	355 (27.9)	243 (30.8)
HIV positive	76 (6.5)	127 (8)	78 (5.8)	42 (4.6)	22 (2.8) *
Parous	707 (60.8)	864 (54.3)	520 (38.5)	433 (47.4)	296 (37.5) *
Nationality					
Italy	1062 (91.3)	1419 (89.1)	1156 (85.7)	774 (84.8)	690 (87.3)
Europe	51 (4.4)	80 (5)	104 (7.7)	78(8.5)	52 (6.6)
Extra-europe	50 (4.3)	93 (5.8)	89(6.6)	61(6.7)	48 (6.1) *
Non-smokers	860 (73.9)	1220 (76.6)	977 (72.4)	692 (75.8)	608 (77)
<10 cig/day	157 (13.5)	185 (11.6)	209 (15.5)	100 (11)	107 (13.5)
≥10 cig/day	146 (12.6)	187 (11.7)	163 (12.1)	121 (13.3)	75 (9.5)
Contraceptive use					
No	544 (46.8)	848 (53.3)	709 (52.6)	526 (57.6)	421 (53.3)
Barrier	112 (9.6)	127 (8)	139 (10.3)	121 (13.3) #	67 (8.5)
Hormonal	482 (41.4)	601 (37.8)	491 (36.4)	257 (28.1) #	300 (38)
IUD	25 (2.1)	16 (1)	10 (0.7)	9 (1)	2 (0.3) *

HIV: Human immunodeficiency Virus; IUD: Intrauterine device; * *p* < 0.05 test for overall linear trend; # *p* < 0.05 by post-hoc chis-quare and Bonferroni correction.

**Table 2:** Colposcopic, virological, cytological, and histological findings in the five periods under study.

	2005–2007 N = 1163 (%)	2008–2010 N = 1592 (%)	2011–2013 N = 1349 (%)	2014–2016 N = 913 (%)	2017–2019 N = 790 (%)
Pap-smear					
ASCUS/LSIL	1007 (86.6)	1452 (91.2)	1197 (88.7)	890 (97.5) #	713 (90.3)
HSIL	156 (13.4) #	140 (8.8)	152 (11.3)	23 (2.5) #	77 (9.7)
Type of virus					
LR-HPV	366 (31.5)	653 (41)	536 (39.7)	167 (18.3) #	124 (15.7) #
HR-HPV vaccine	542 (46.6)	631 (39.6)	488 (36.2) #	440 (48.2) #	301 (38.1)
HR-HPV others	93 (8) #	216 (13.6)	196 (14.5)	145 (15.9)	141 (17.8) #
Negative	105 (9)	83 (5.2) #	81 (6) #	96 (10.5)	108 (13.7) #
Untypable	57 (4.9)	9 (0.6) #	48 (3.6)	65 (7.1)	116 (14.7) #*
Multiple HPV infection:	471 (40.5)	813 (51.1)	663 (49.1)	156 (17.1)	133 (16.8) *
Colposcopic lesion >50%	193 (16.59)	236 (14.9)	309 (22.9)	101 (11.1)	199 (21.8) *
Excisional cervical treatment	268 (23)	357 (22.4)	244 (18.1)	157 (17.2)	138 (17.5) *
**Histology**	**2005–2007** **N = 693 (%)**	**2008–2010** **N = 934 (%)**	**2011–2013** **N = 739 (%)**	**2014–2016** **N = 525 (%)**	**2017–2019** **N = 513 (%)**
Negative	240 (34.6) #	274 (29.3)	179 (24.2)	96 (18.3) #	104 (20.3) #
CIN1	269 (38.8) #	411 (44)	346 (46.8)	282 (53.7)	258 (50.3)
CIN2	47 (6.8) #	85 (9.1)	70 (9.5)	70 (13.3)	73 (14.2)
CIN3	124 (17.9)	149 (16)	128 (17.3)	70 (13.3)	75 (14.6)
Squamous Invasive Cancer	13 (1.9)	15 (1.6)	16 (2.2)	7 (1.3)	3 (0.6) *
Multiple HPV infection:	471 (40.5)	813 (51.1)	663 (49.1)	156 (17.1)	133 (16.8) *

ASCUS: Atypical Squamous Cells of Undetermined Significance; LSIL: Low-grade squamous intraepithelial lesion; HSIL: High-grade squamous intraepithelial lesion; LR-HPV: Low-Risk Human Papillomavirus; HR-HPV: High Risk Human Papillomavirus; CIN: cervical intraepithelial Neoplasia. * *p* < 0.05 test for overall linear trend; # *p* < 0.05 by post-hoc chi-square and Bonferroni correction.

**Table 3:** Association between cytological findings et enrolment and HR-HPV genotypes contained in Nonavalent vaccine in five periods under study.

		2005–2007 N = 693 (%)	2008–2010 N = 934 (%)	2011–2013 N = 739 (%)	2014–2016 N = 525 (%)	2017–2019 N = 513 (%)
Negative/CIN1	HPV16	125 (18)	135 (14.5)	114 (15.4)	78 (14.9)	39 (7.6) *
	HPV18	37 (5.3)	27 (2.9)	21 (2.8)	22 (4.2)	16 (3.1)
	HPV31	89 (12.8)	48 (5.1)	25 (3.4)	48 (9.1)	25 (4.9) *
	HPV33	13 (1.9)	18 (1.9)	13 (1.8)	18 (3.4)	4 (0.8)
	HPV45	10 (1.4)	11 (1.2)	6 (0.8)	10 (1.9)	5 (1)
	HPV52	53 (7.6)	145 (15.5)	96 (13)	64 (12.2)	29 (5.7)
	HPV 58	9 (1.3)	8 (0.9)	9 (1.2)	20 (3.8)	24 (4.7)
CIN2+	HPV16	80 (11.5)	85 (9.1)	77 (10.4)	65 (12.4)	56 (10.9)
	HPV18	42 (6.1)	27 (2.9)	6 (0.8)	11 (2.1)	9 (1.8) *
	HPV31	57 (8.2)	39 (4.2)	33 (4.5)	22 (4.2)	20 (3.9) *
	HPV33	7 (1)	23 (2.5)	10 (1.4)	11 (2.1)	11 (2.1)
	HPV45	15 (2.2)	7 (0.7)	2 (0.3)	7 (1.3)	7 (1.4)
	HPV52	47 (6.8)	79 (8.5)	67 (9.1)	14 (2.7)	15 (2.9) *
	HPV 58	6 (0.9)	7 (0.7)	3 (0.4)	10 (1.9)	6 (1.2)

ASCUS: Atypical Squamous Cells of Undetermined Significance; LSIL: Low-grade squamous intraepithelial lesion; HSIL: High-grade squamous intraepithelial lesion; HR-HPV: High Risk Human Papillomavirus. * *p* < 0.05 test for overall linear trend.

**Table 4:** Association between histological findings et enrolment and HR-HPV genotypes of Nova-valet vaccine in the five periods under study.

		2005–2007 N = 693 (%)	2008–2010 N = 934 (%)	2011–2013 N = 739 (%)	2014–2016 N = 525 (%)	2017–2019 N = 513 (%)
Negative/CIN1	HPV16	125 (18)	135 (14.5)	114 (15.4)	78 (14.9)	39 (7.6) *
	HPV18	37 (5.3)	27 (2.9)	21 (2.8)	22 (4.2)	16 (3.1)
	HPV31	89 (12.8)	48 (5.1)	25 (3.4)	48 (9.1)	25 (4.9) *
	HPV33	13 (1.9)	18 (1.9)	13 (1.8)	18 (3.4)	4 (0.8)
	HPV45	10 (1.4)	11 (1.2)	6 (0.8)	10 (1.9)	5 (1)
	HPV52	53 (7.6)	145 (15.5)	96 (13)	64 (12.2)	29 (5.7)
	HPV58	9 (1.3)	8 (0.9)	9 (1.2)	20 (3.8)	24 (4.7)
CIN2+	HPV16	80 (11.5)	85 (9.1)	77 (10.4)	65 (12.4)	56 (10.9)
	HPV18	42 (6.1)	27 (2.9)	6 (0.8)	11 (2.1)	9 (1.8) *
	HPV31	57 (8.2)	39 (4.2)	33 (4.5)	22 (4.2)	20 (3.9) *
	HPV33	7 (1)	23 (2.5)	10 (1.4)	11 (2.1)	11 (2.1)
	HPV45	15 (2.2)	7 (0.7)	2 (0.3)	7 (1.3)	7 (1.4)
	HPV52	47 (6.8)	79 (8.5)	67 (9.1)	14 (2.7)	15 (2.9) *
	HPV58	6 (0.9)	7 (0.7)	3 (0.4)	10 (1.9)	6 (1.2)

CIN: Cervical Intraepitelial Neoplasia; HR-HPV: High Risk Human Papillomavirus.* *p* < 0.05 test for overall linear trend.

## Data Availability

The data presented in this study are available on request to the corresponding author.
